# Association between Duration of Folic Acid Supplementation during Pregnancy and Risk of Postpartum Depression

**DOI:** 10.3390/nu9111206

**Published:** 2017-11-02

**Authors:** Jing Yan, Yuyan Liu, Lujia Cao, Yuzhi Zheng, Wen Li, Guowei Huang

**Affiliations:** 1 Department of Nutrition and Food Science, School of Public Health, Tianjin Medical University, 22 Qixiangtai Road, Heping District, Tianjin 300070, China; yanjing@tmu.edu.cn (J.Y.); 586324lyy@tmu.edu.cn (Y.L.); caolujia@tmu.edu.cn (L.C.); zhengyuzhi@tmu.edu.cn (Y.Z.); liwen828@tmu.edu.cn (W.L.); 2 Department of Social Medicine and Health Administration, School of Public Health, Tianjin Medical University, 22 Qixiangtai Road, Heping District, Tianjin 300070, China

**Keywords:** folic acid, supplementation duration, postpartum depression, propensity score matching

## Abstract

Postpartum depression (PPD), as a common complication of childbearing, could have adverse consequences on mothers, children, and families. This cohort study aimed to assess the association between duration of folic acid (FA) supplementation during pregnancy and the onset of PPD in Chinese women. A total of 1592 participants were recruited, and data collected between July 2015 and March 2017 in Tianjin, China. Participants’ baseline data were collected regarding socio-demographic and lifestyle characteristics, obstetric history, and FA supplementation during pregnancy. The Chinese version of the self-rating depression scale was used to assess depressive symptoms at 6–12 weeks postpartum, and the prevalence of PPD in participants was 29.4%. Pregnant women who took FA supplements for >6 months had a lower prevalence of PPD, compared to those who took FA for ≤6 months. After using the 1:1 ratio propensity score matching, 601 FA-users ≤ 6 months and 601 FA-users > 6 months were included in the further analyses; this also yielded similar results (*P* < 0.05). Logistic regression analysis showed that FA intake for >6 months was an independent determinant of PPD (odds ratio = 0.76; 95% confidence interval: 0.59–0.98; *P* < 0.05). Thus, prolonged FA supplementation during pregnancy was associated with a decreased risk of PPD in Chinese women.

## 1. Introduction

Depression is a major mental health problem and is the leading cause of disability worldwide [1,2]. According to the World Health Organization (WHO), more than 300 million people suffer from depression worldwide, and it is more common among women than men [1]. Postpartum depression (PPD), as a common complication of childbearing, is defined as an obvious depressive symptom or a typical depressive episode within 1 to 12 months after delivery [2,3,4]. Due to differences in assessment tools, research methods, survey time points after childbirth, and countries of study samples, the prevalence rate of PPD varies widely worldwide. Previous systemic reviews reported that the prevalence of PPD ranges from 0% to 60% based on 40 countries in 2002 [5], and from 3.5% to 63% based on 17 Asian countries between 1998 and 2008 [6]. The symptoms of PPD include reduced interest, poor concentration, sleep deprivation, altered eating pattern, sadness, hopelessness, anxiety, and irritability [1,2,3,7]. Thus, PPD constitutes an adverse consequence for mothers, children, and families [2,3,7,8,9]. For mothers, PPD is associated with adverse health outcomes (e.g., mental impairment, chronicity of the depression, appetite disorder, decline in sleep quality, and even suicide), and adverse behavioural outcomes (e.g., less responsive caregiving, reduced breastfeeding, less safety concern for their children, rejection of their children, abusive behaviour, and infanticide) [7,8,9]. Children of mothers with PPD have higher a risk of negative consequences in social, emotional, cognitive, and developmental areas, such as sleep disturbances, irritability, negative emotionality, poorer physical growth, infant malnutrition, diarrhoea, and respiratory disease [7,8,9]. For families, PPD could cause tension in marital relationships and breakdown of marriages [7,8,9]. PPD also increases the risk for long-term cognitive impairment, emotional difficulties, and behavioural problems for mothers and children [2,3].

At present, the aetiology of PPD is unknown, but it is generally believed to be linked to biological, genetic, hormonal, psychosocial, and environmental factors [1,2,3,4,7,8,9,10]. In addition, nutritional factors have been identified as determinants of PPD, such as folate/folic acid (FA), vitamin B-12, calcium, iron, selenium, zinc, and polyunsaturated fatty acids [3,8,11]. Although there are numerous studies examining the relationship between nutrients and PPD, a limited number of investigations assess the link between folate and PPD. However, studies have reported that an adequate level of folate may reduce the risk of depression in the general population [12,13,14,15]. Furthermore, previous studies have investigated the mechanism of folate on depression in the general population. Folate is essential for the biosynthesis of the monoamine neurotransmitters (e.g., serotonin, dopamine, and norepinephrine). Folate participates in the production of *S*-adenosylmethionine (SAM) through homocysteine (Hcy) remethylation, and SAM is essential for the production of these three aforementioned neurotransmitters [3,16]. Lack of folate could inhibit the transformation of Hcy to cysteine, thereby increasing the plasma levels of Hcy [12,13]. Several studies have shown Hcy levels to be positively correlated with the severity of depression [12,13,14,15,16]. Meanwhile, pregnant women require more intake of folate/FA compared to non-pregnant women, and there is a high prevalence of inadequate folate/FA intake among pregnant women [11,17]. According to the mechanism of folate on depression, inadequate folate/FA intake during pregnancy may lead to PPD [3,11].

Currently, PPD is a common and serious mental disorder, and there is no effective treatment [2,18]. Therefore, it is of great practical significance to explore the prevention of PPD. Although previous studies have identified that adequate levels of folate prevent the onset of depression in the adolescent, adult, and elderly population, pregnant women are excluded as a special population [3,12]. Moreover, few studies associate folate deficiency with a high risk of PPD [3,11], but to the best of our knowledge, none of these focused on the link between FA supplementation during pregnancy and PPD in Chinese women. The FA supplementation is an effective way to protect against folate deficiency, and thus, it is important to explore an appropriate way of FA supplementation, in order to prevent PPD. Furthermore, the onset of PPD between 6 and 12 weeks after giving birth is most often in the first postpartum year [3,19], but no study addresses the association between FA supplementation during pregnancy and PPD at 6–12 weeks postpartum.

This study is one of few to examine the effect of the duration of FA supplementation during pregnancy on PPD in Chinese women, and thus, may provide new information regarding the potential beneficial effect of long term FA supplementation during pregnancy. The purpose of this investigation is to assess the association between the duration of FA supplementation during pregnancy, and the onset of PPD 6–12 weeks after delivery in Chinese women.

## 2. Materials and Methods

### 2.1. Population

**Figure 1:**
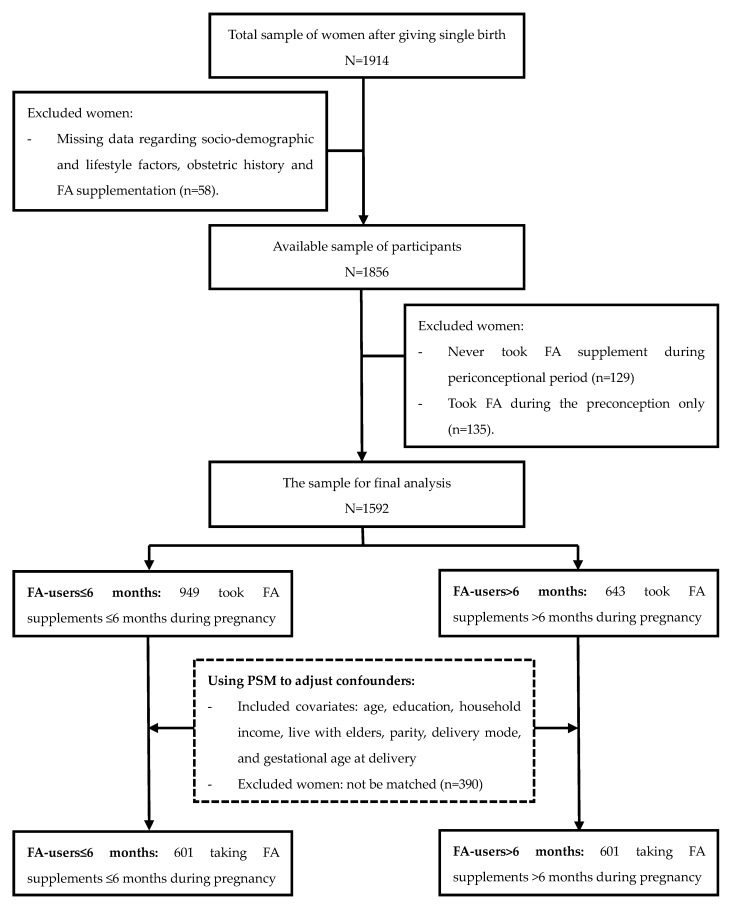
Flow diagram of enrolment in the study. FA: folic acid; PSM: propensity score matching.

This cohort study recruited participants at 6–12 weeks postpartum, who had their first postnatal check-ups at two maternal and child healthcare centres in Tianjin, China, between July 2015 and March 2017. Questionnaires were used to collect participant information regarding socio-demographic and lifestyle characteristics, obstetric history, FA supplementation, and depressive symptoms, by face-to-face interviews. A total of 1914 women, who delivered a singleton foetus, were recruited in this study, while 322 of them were excluded: 58 women had missing data regarding socio-demographic and lifestyle factors, obstetric history, and FA supplementation; 129 women did not use FA during pregnancy; and 135 women used FA in the preconceptional period only. Finally, 1592 participants who took FA supplements during pregnancy were involved in the further analysis (Figure 1). All participants provided informed consent in writing. The protocol of this study was approved by the Ethics Committee of the Tianjin Medical University.

### 2.2. Depressive Symptoms

The Chinese version of the Self-Rating Depression Scale (SDS) was used to assess depressive symptoms. There were 20 items in the SDS, including 10 positive items and 10 negative items. Each item was scored on 4-point scale to evaluate the frequency of symptoms: “1” none or a little time; “2” a small part of the time; “3” a lot of time; “4” most of the time. The raw score was a sum of the scores from the 20 items, which was multiplied by 1.25 to yield the standard score [20,21]. Based on the standard score, a cut-off point of 50 was used to define depression symptoms among the participants: no depression (<50), and depression (≥50) [21,22,23]. Furthermore, a higher score reflected more severe symptoms of PPD.

### 2.3. Folic Acid Supplementation

A self-reported questionnaire was used to collect the information on FA supplementation during pregnancy, including the supplement brand name, the time of supplement initiation and termination, intake duration, frequency, and dose. Participants were assessed about their FA supplementation status during pregnancy by using their recall response. FA users included those who used FA supplements, as well as those who used FA-containing multivitamins. Among 1592 participants, 803 (50.4%) participants took FA supplements during pregnancy for a duration of 3 months or less, 146 (9.2%) took FA supplements during pregnancy for a duration 4–6 months, and 643 (40.4%) took FA supplements during pregnancy for a duration of more than 6 months. According to the feature of the FA supplementation’s duration in this cohort, participants were divided into two groups: FA-users ≤ 6 months and FA-users > 6 months.

### 2.4. Covariates

A general health questionnaire was used to collect information on demographic characteristics, lifestyle, and obstetric history. Education level was classified into three categories: ≤12 years, 13–16 years, and >16 years. Household income, which was the total monthly income for all family members, was divided into three categories: <5000 Chinese Yuan (CNY), 5000–10,000 CNY, and >10,000 CNY. The variable “live with parents” (own parents and/or parents-in-law) was classified into two categories: yes and no. Parity was divided into two categories: primipara (no previous children) and multipara (≥1 previous child). Delivery mode included vaginal birth and caesarean birth, and gestational age at delivery was divided into two categories (<37 weeks and ≥37 weeks). 

### 2.5. Statistical Analysis

All analyses were conducted using the Statistical Package for the Social Sciences software version 22.0 (SPSS Inc., Chicago, IL, USA). The chi-squared test and independent-sample t test was used to assess differences in participants’ characteristics and depressive symptoms stratified between FA-users ≤ 6 months and FA-users > 6 months. Propensity score matching (PSM) was used to minimise the effects of confounding variables related to differences in the two groups of participants (FA-users ≤ 6 months and FA-users > 6 months), in order to assess the association between duration of FA supplementation and the onset of PPD. Propensity is the probability of inclusion into the two groups (FA-users ≤ 6 months and FA-users > 6 months) depending on the respective participants’ characteristics. The propensity score is the predicted probability for each participant from a logistic regression model with the duration of FA usage as the dependent variable and all the participant characteristics as independent variables [24]. Propensity scoring generates a single score based on the observed covariate data to effectively match women from different groups. In this study, propensity scores were calculated by using a logistic regression model and the following covariates: age, education, household income, live with elders, parity, delivery mode, and gestational age at delivery. Thus, the distributions of characteristics were similar between FA-users ≤ 6 months and FA-users > 6 months. We did a 1:1 nearest neighbour matching with a caliper distance of 0.02. The caliper is a preset range of PSM. When the difference of propensity score between subjects in two groups is within this range, these two subjects could be matched. Common values of caliper are 0.005, 0.01, 0.02, 0.03, and 0.1 [25]. According to the results of PSM, a total of 1202 women (601 FA-users ≤ 6 months & 601 FA-users > 6 months) were involved in the further analysis (Figure 1). The association between the duration of FA supplementation and PPD would be examined by comparing the matched data. Pearson’s chi-squared test and independent-sample t test was used to compare PPD prevalence rates between two propensity score-matched groups. Finally, logistic regression analysis was used to estimate the odds ratio (OR) and 95% confidence interval (CI), in order to further confirm the findings of the PSM. Age, education, household income, live with parents, parity, delivery mode, and gestational age at delivery were evaluated for inclusion in the logistic regression model. *P*-values < 0.05 was considered statistically significant.

## 3. Results

A total of 1592 women were enrolled in this study, and the mean age of participants was 31.03 years (standard deviation (SD) = 3.81, age range: 21–43 years). Approximately 90% of participants had post-secondary education, and 68% of the women earned a household income of 10,000 CNY and less. Approximately 30% of participants lived with their parents, including their own parents and/or parents-in-law. More than 70% of the women were primiparous, and over half of the infants were delivered via caesarean birth. Only 5.4% of the infants were premature. Among the 1592 FA-users, 643 (40.4%) took FA supplements during pregnancy for a duration of greater than six months. Women with FA supplementation >6 months were older, had higher education levels, and higher household income (*P* < 0.05) (Table 1). Among these 1592 women, 29.4% were identified as having PPD. The prevalence of PPD was significantly higher among participants who reported taking FA for a duration of 6 months or less during pregnancy than those who reported taking FA for a duration of more than 6 months during pregnancy (*P* < 0.05) (Table 1).

According to results of PSM, a total of 1202 women were involved in the further analysis. After adjusting for propensity scores in the current analysis, there were no significant differences in the baseline characteristics of the women in both groups (FA-users ≤ 6 months and FA-users > 6 months) (*P* > 0.05). The prevalence of PPD decreased from 32.2% to 30.6% among women with a period of FA intake of 6 months or less, and increased from 25.2% to 25.3% among women with a period of FA intake of more than 6 months; these differences in both groups were statistically significant (*P* < 0.05) (Table 2).

From the results of the logistic regression analysis, the intake of FA for more than 6 months was an independent determinant of PPD, with an OR of 0.76 (95% CI: 0.59–0.98, *P* < 0.05) (Table 3). Thus, FA supplementation for more than 6 months during pregnancy was associated with a decreased risk of PPD.

## 4. Discussion

Depression is one of the most common and severe mental disorders worldwide, with a prevalence rate in females 2- to 3-times higher than in males [1,3,16]. It has been indicated that the incidence of depression is high during puberty, as well as the premenstrual, periconceptional, and menopausal periods [1,3]. Particularly, PPD as a special form of depression has become a significant public concern. Numerous previous studies have reported prevalence rates of PPD in women from different countries. According to study results in Chinese women, the incidence rate ranged from 6.5% to 30% [7,9,26,27]. Several studies have reported that the prevalence rate of PPD in Canadian, Malaysian, American, Vietnamese, and Korean women is 8.0% [28], 14.3% [29], 15.4% [30], 19.3% [4], and 36.3–36.7% [31], respectively. The present findings show that the prevalence rate of PPD is 29.4% in women who took FA supplements during pregnancy. In present study, women, who did not take FA supplements during pregnancy and took FA supplements in the preconceptional period only, were excluded. The reasons are as follows. Firstly, the aims of this study are to assess the association between the duration of FA supplementation during pregnancy and the onset of PPD 6–12 weeks after delivery in Chinese women. Secondly, these are not common phenomenon in China, that women do not take FA supplements or took FA supplements in the preconception only. The Chinese government places a high priority on promoting the prevalence of periconceptional FA supplementation. In China, reproductive women are recommended to take 400 μg/day of FA supplementation from three months preconception until the end of the first trimester of pregnancy by the National Health and Family Planning Commission [32]. Moreover, free FA supplements have been provided to reproductive women living in rural areas by a national project “folic acid supplementation to prevent neural tube defects” since 2009 [32]. Therefore, small sample sizes of women who never used FA supplements, or use FA supplements in the preconception only, are generated. If women who never used FA supplements or used FA supplements in the preconception only are included in this study, the unbalanced sample size will influence the statistical power of results. Thus, this study focuses on women who took FA supplements during pregnancy.

According to previous studies worldwide, the determinants of PPD have included demographic factors, socio-economic status, family history and social support, environmental and cultural factors, nutrients, inflammatory factors, hormones, and biological factors [1,2,3,4,5,6]. Several investigations reported that folate level was associated with cognitive function and depression in the general population [12,13,14,15], while few previous studies focused on the relationship between folate and PPD [11,16,33,34,35]. However, the results of studies on the relationship between FA supplementation during pregnancy and the onset of PPD are conflicting. FA supplementation during pregnancy has been identified as a protective factor for PPD in some studies [11,16], while others failed to establish a link between FA supplementation and PPD [33,34,35]. For example, Lewis et al. [11] reported that women who took FA supplementation during pregnancy had lower scores for depression 21 months postpartum, compared to women who did not take supplements. Behzadi et al. [16] also identified FA as preventing the onset of antepartum depression and PPD in a previous study. In contrast, a cohort study in Singapore showed there was no difference in maternal folate levels between participants with and without PPD [33]. Miyake et al. [34] found no association between dietary FA intake during pregnancy and PPD in Japanese women with inadequate folate intake. Blunden et al. [35] reported that red blood cell folate concentrations, which were measured before pregnancy and during the first trimester, were not associated with PPD. In this previous study, the average time interval between the measurement of red-cell folate during pregnancy and screening for PPD was one year; hence, folate levels in the postpartum period were not consistent with the folate level in early pregnancy. Significantly, investigations regarding the association between the duration of FA supplementation during pregnancy and the onset of PPD are limited. According to a previous review involving 36 countries, the recommended duration of FA supplementation during pregnancy varies for different countries; however, no consensus has been reached regarding this issue [36]. The aim of this study was to examine the association between the duration of FA supplementation during pregnancy and the onset of PPD, in order to provide useful information regarding appropriate duration of FA supplementation during pregnancy. The present findings show that long-term FA supplementation (>6 months) during pregnancy is significantly associated with a lower prevalence of PPD. This result not only demonstrates the relationship between FA supplementation during pregnancy and PPD, but also emphasises the importance of long-term use in order to ensure that FA reaches significant levels in the body to activate physiological function.

Regarding socio-demographic factors linked to PPD, existing studies have assessed predictors of socio-economic status, demographic factors, lifestyle, and obstetric history [7,8,9,10]. Based on previous literature, populations with higher prevalence of PPD were more likely to be women with lower education levels, lower household income, poor relationship with family members (e.g., husbands, parents or parents-in-law), primiparous, who had undergone caesarean delivery, and delivered premature babies [7,8,9,10]. Furthermore, previous studies have also reported that FA supplementation during pregnancy could be influenced by childbearing age, maternal education level, household income, and parity [37,38,39]. Women who reported using FA supplements for a longer duration tend to be older, with higher education level, with higher family income, and primiparous [37,38,39]. Therefore, the present study factored in covariates into the analysis, including age, education, household income, living with parents, parity, delivery mode, and gestational age at delivery. The present results showed that women with FA supplementation for more than 6 months during pregnancy tended to be older, had higher education levels, and higher household income (*P* < 0.001). Thus, PSM was used to minimise the effects of confounding variables related to differences in the two groups of participants (FA-users ≤ 6 months and FA-users > 6 months), in order to assess the association between the duration of FA supplementation during pregnancy and the onset of PPD. PSM is widely used in statistical analyses, and can effectively match participants from different groups [24,40]. To the best of our knowledge, this study is the first to apply PSM in evaluating the association between the duration of FA supplementation during pregnancy and the onset of PPD. After using PSM, the two groups (FA-users ≤ 6 months and FA-users > 6 months) were comparable after controlling for multiple variables, including age, education, household income, living with parents, parity, delivery mode, and gestational age at delivery. The results after adjusting for propensity scores showed that longer durations of FA supplementation during pregnancy were associated with a lower prevalence of PPD. The logistic regression analysis also yielded similar results.

The main strength of this study is that it provided useful information regarding the effect of the duration of FA intake during pregnancy on the onset of PPD. It demonstrated that populations with FA usage more than 6 months during pregnancy had a lower PPD prevalence. Also, this study included a large number of participants, and balanced the confounding factors more efficiently by using PSM, which could reduce the level of bias due to confounding variables [24,40]. PSM could include more confounders, be a better solution of eliminating collinearities, and obtain an estimate of treatment effect which is closer to the true average treatment effect, compared with logistic regression model [41]. Thus, the present findings were more objective and accurate.

There is no denying that this study has a few limitations. First, recall bias was possible, because the data on FA supplementation was collected 6–12 weeks after giving birth. The face-to-face interviews were conducted by professional and well-trained investigators, in order to obtain more accurate information. Second, this study investigated FA supplementation, but dietary folate and serum folate levels were not measured. The serum folate levels depend on dietary folate and FA supplementation. Serum levels of folate and Hcy should be assessed in future studies. Third, the SDS was used as a screening tool, but clinical diagnoses were lacking. Although SDS scores are inconsistent with clinical diagnoses, the total scores showed depressive symptoms to be related to clinical outcomes [22]. Moreover, the SDS was designed for the general population, and may be less sensitive during the screening of pregnant women. However, previous literatures have confirmed that the SDS can be used to evaluate depression among women post-delivery [42,43,44]. Finally, although several covariates were included in the matching process, more confounding factors should be assessed in future research. For example, antenatal depression and PPD might be a continuum reflecting an underlying chronic condition among women during pregnancy and thereafter [3,7,22]. The unmeasured factors may have an impact on the observed association. Therefore, we suggest that future studies could assess these unmeasured confounding factors further. 

## 5. Conclusions

This study showed a high prevalence rate of PPD among Chinese women. FA supplementation for more than 6 months during pregnancy was found to be associated with a decreased risk of PPD 6–12 weeks after delivery in Chinese women. The finding provides new information regarding the potential beneficial effect of long-term FA usage during pregnancy.

## Figures and Tables

**Table 1:** Characteristics of study participants and prevalence of PPD according to the duration of FA supplementation.

Characteristics	FA-Users ≤ 6 Months	FA-Users > 6 Months	*P* ^a^
	*n* = 949	*n* = 643	
Age (*years*, *mean* ± *SD*)	30.7 ± 4.0	31.5 ± 3.5	<0.001
Education (*years*, *n* (%))			<0.001
≤12	132 (13.9%)	33 (5.1%)	
13–16	721 (76.0%)	486 (75.6%)	
>16	96 (10.1%)	124 (19.3%)	
Household income (*CNY*, *n* (%))			<0.001
<5000	263 (27.7%)	97 (15.1%)	
5000–10,000	443 (46.7%)	280 (43.5%)	
>10,000	243 (25.6%)	266 (41.4%)	
Live with parents (*n* (%))			0.554
Yes	266 (28.0%)	189 (29.4%)	
No	683 (72.0%)	454 (70.6%)	
Parity (*n* (%))			0.323
Primipara	657 (69.2%)	460 (71.5%)	
Multipara	292 (30.8%)	183 (28.5%)	
Delivery mode (*n* (%))			0.854
Vaginal birth	462 (48.7%)	310 (48.2%)	
Caesarean birth	487 (51.3%)	333 (51.8%)	
Gestational age at delivery (*weeks*, *n* (%))			0.195
<37	57 (6.0%)	29 (4.5%)	
≥37	892 (94.0%)	614 (95.5%)	
Prevalence of PPD (*n* (%))			0.002
No depression ^b^	643 (67.8%)	481 (74.8%)	
Depression ^b^	306 (32.2%)	162 (25.2%)	

PPD: postpartum depression; FA: folic acid; CNY: Chinese Yuan; ^a^ Analysis using a chi-squared test and independent-sample *t* test; ^b^ Women were divided into two groups according to self-rating depression scale scores, <50 for no depression, ≥50 for depression.

**Table 2:** Characteristics of study participants and prevalence of PPD according to the duration of FA supplementation after propensity matching (*n* (%)).

Characteristics	FA-Users ≤ 6 Months	FA-Users > 6 Months	*P* ^a^
	*n* = 601	*n* = 601	
Age (*years, mean ± SD*)	31.7 ± 3.8	31.4 ± 3.5	0.107
Education (*years, n (%)*)			0.731
≤12	37 (6.2%)	33 (5.5%)	
13–16	470 (78.2%)	481 (80.0%)	
>16	94 (15.6%)	87 (14.5%)	
Household income (*CNY, n (%)*)			0.279
<5000	117 (19.5%)	97 (16.1%)	
5000–10,000	276 (45.9%)	279 (46.4%)	
>10,000	208 (34.6%)	225 (37.5%)	
Live with parents (*n* (%))			0.610
Yes	168 (28.0%)	176 (29.3%)	
No	433 (72.0%)	425 (70.7%)	
Parity (*n* (%))			0.344
Primipara	414 (68.9%)	429 (71.4%)	
Multipara	187 (31.1%)	172 (28.6%)	
Delivery mode (*n* (%))			0.526
Vaginal birth	302 (50.2%)	291 (48.4%)	
Caesarean birth	299 (49.8%)	310 (51.6%)	
Gestational age at delivery (*weeks, n (%)*)			0.791
<37	31 (5.2%)	29 (4.8%)	
≥37	570 (94.8%)	572 (95.2%)	
Prevalence of PPD (*n* (%))			0.040
No depression ^b^	417 (69.4%)	449 (74.7%)	
Depression ^b^	184 (30.6%)	152 (25.3%)	

PPD: postpartum depression; FA: folic acid; CNY: Chinese Yuan; ^a^ Analysis using a chi-squared test and independent-sample *t* test; ^b^ Women were divided into two groups according to self-rating depression scale scores, <50 for no depression, ≥50 for depression.

**Table 3:** The association between the duration of FA supplementation during pregnancy and risk of PPD.

Duration of FA Supplementation	OR ^a^	95% CI	*P* ^b^
≤6 months	1		
>6 months	0.76	0.59–0.98	0.037

FA: folic acid; PPD: postpartum depression; OR: odds ratio; CI: confidence interval; ^a^ Adjusted for age, education, household income, live with elders, parity, delivery mode, and gestational age at delivery; ^b^ Analysis using a multivariable logistic regression model.

## Abbreviations

The following abbreviations are used in this manuscript:

CI	confidence interval
CNY	Chinese Yuan
FA	folic acid
Hcy	homocysteine
OR	odds ratio
PPD	postpartum depression
PSM	propensity score matching
SAM	*S*-adenosylmethionine
SD	standard deviation
SDS	self-rating depression scale
WHO	World Health Organization

## References

[1] World Health Organization Depression. http://www.who.int/mediacentre/factsheets/fs369/en/.

[2] Sockol L.E., Epperson C.N., Barber J.P. (2013). Preventing postpartum depression: A meta-analytic review. Clin. Psychol. Rev..

[3] Leung B.M., Kaplan B.J. (2009). Perinatal depression: Prevalence, risks, and the nutrition link—A review of the literature. J. Am. Diet. Assoc..

[4] Van Vo T., Hoa T.K.D., Hoang T.D. (2017). Postpartum depressive symptoms and associated factors in married women: A Cross-sectional study in Danang City. Front. Public Health.

[5] Miller L.J. (2002). Postpartum depression. JAMA.

[6] Klainin P., Arthur D.G. (2009). Postpartum depression in Asian cultures: A literature review. Int. J. Nurs. Stud..

[7] Ding T., Wang D.X., Qu Y., Chen Q., Zhu S.N. (2014). Epidural labor analgesia is associated with decreased risk of postpartum depression: A prospective cohort study. Anesth. Analg..

[8] Aishwarya S., Rajendiren S., Kattimani S., Dhiman P., Haritha S., AnanthaNarayanan P.H. (2013). Homocysteine and serotonin: Association with postpartum depression. Asian J. Psychiatry.

[9] Chi X., Zhang P., Wu H., Wang J. (2016). Screening for postpartum depression and associated factors among women in China: A cross-sectional study. Front. Psychol..

[10] Deng A.W., Xiong R.B., Jiang T.T., Luo Y.P., Chen W.Z. (2014). Prevalence and risk factors of postpartum depression in a population based sample of women in Tangxia community, Guangzhou. Asian Pac. J. Trop. Med..

[11] Lewis S.J., Araya R., Leary S., Smith G.D., Ness A. (2012). Folic acid supplementation during pregnancy may protect against depression 21 months after pregnancy, an effect modified by MTHFR C677T genotype. Eur. J. Clin. Nutr..

[12] Beydoun M.A., Shroff M.R., Beydoun H.A., Zonderman A.B. (2010). Serum folate, vitamin B-12, and homocysteine and their association with depressive symptoms among U.S. adults. Psychosom. Med..

[13] Pan W.H., Chang Y.P., Yeh W.T., Guei Y.S., Lin B.F., Wei L.L., Yang F.L., Liaw Y.P., Chen K.J., Chen W.J. (2012). Co-occurrence of anemia, marginal vitamin B6, and folate status and depressive symtoms in older adults. J. Geriatr. Psychiatry Neurol..

[14] Bjelland I., Ueland P.M., Vollset S.E. (2003). Folate and depression. Psychother. Psychosom..

[15] Ramos M.I., Allen L.H., Haan M.N., Green R., Miller J.W. (2004). Plasma folate concentrations are associated with depressive symptoms in elderly Latina women despite folic acid fortification. Am. J. Clin. Nutr..

[16] Behzadi A.H., Behbahani A.S., Ostovar N. (2008). Therapeutic effects of folic acid on ante partum and postpartum depression. Med. Hypotheses.

[17] Wang S., Ge X., Zhu B., Xuan Y., Huang K., Rutayisire E., Mao L., Huang S., Yan S., Tao F. (2016). Maternal Continuing Folic Acid Supplementation after the First Trimester of Pregnancy Increased the Risk of Large-for-Gestational-Age Birth: A Population-Based Birth Cohort Study. Nutrients.

[18] Liu S., Yan Y., Gao X., Xiang S., Sha T., Zeng G., He Q. (2017). Risk factors for postpartum depression among Chinese women: Path model analysis. BMC Pregnancy Childbirth.

[19] Gavin N.I., Gaynes B.N., Lohr K.N., Meltzer-Brody S., Gartlehner G., Swinson T. (2005). Perinatal depression: A systematic review of prevalence and incidence. Obstet. Gynecol..

[20] Hunter E.E., Murphy M. (2014). Zung self-rating depression scale. Encycl. Clin. Neuropsychol..

[21] Zung W.W. (1965). A Self-Rating Depression Scale. Arch. Gen. Psychiatry.

[22] Zung W.W., Richards C.B., Short M.J. (1965). Self-rating depression scale in an outpatient clinic. Further validation of the SDS. Arch. Gen. Psychiatry.

[23] Guo N.F., Yu J.S. (2012). National Vocational Qualification Training Course Psychological Consultant (Level 3).

[24] Rosenbaum P., Rubin D. (1983). The central role of the propensity score in observational studies for casual effects. Biometrika.

[25] Austin P.C. (2009). Some methods of propensity-score matching had superior performance to others: Results of an empirical investigation and Monte Carlo simulations. Biom. J..

[26] Mao Q., Zhu L.X., Su X.Y. (2011). A comparison of postnatal depression and related factors between Chinese new mothers and fathers. J. Clin. Nurs..

[27] Xie R.H., He G., Liu A., Bradwejn J., Walker M., Wen S.W. (2007). Fetal gender and postpartum depression in a cohort of Chinese women. Soc. Sci. Med..

[28] Dennis C.L., Heaman M., Vigod S. (2012). Epidemiology of postpartum depressive symptoms among Canadian women: Regional and national results from a cross-sectional survey. Can. J. Psychiatry.

[29] Yusuff A.S.M., Tang L., Binns C.W., Lee A.H. (2015). Prevalence and risk factors for postnatal depression in Sabah, Malaysia: A cohort study. Women Birth.

[30] Halbreich U., Karkun S. (2006). Cross-cultural and social diversity of prevalence of postpartum depression and depressive symptoms. J. Affect. Disord..

[31] Youn J.H., Jeong I.S. (2013). Predictors of postpartum depression: Prospective cohort study. J. Korean Acad. Nurs..

[32] National Health and Family Planning Commission of the People’s Republic of China The Project Management Plan of Folic Acid Supplement to Prevent Neural Tube Defects. http://www.nhfpc.gov.cn/fys/s3581/201006/942109bebb4340b2922898f565489a6f.shtml.

[33] Chong M.F.F., Wong J.X.Y., Colega M., Chen L.W., van Dam R.M., Tan C.S., Lim A.L., Cai S., Broekman B.F.P., Lee Y.S. (2014). Relationships of maternal folate and vitamin B12 status during pregnancy with perinatal depression: The GUSTO study. J. Psychiatry Res..

[34] Miyake Y., Sasaki S., Tanaka K., Yokoyama T., Ohya Y., Fukushima W., Saito K., Ohfuji S., Kiyohara C., Hirota Y. (2006). Dietary folate and vitamins B12, B6, and B2 intake and the risk of postpartum depression in Japan: The Osaka Maternal and Child Health Study. J. Affect. Disord..

[35] Blunden C.H., Inskip H.M., Robinson S.M., Cooper C., Godfrey K.M., Kendrick T.R. (2012). Postpartum depressive symptoms: The B-vitamin link. Ment. Health Fam. Med..

[36] Gomes S., Lopes C., Pinto E. (2016). Folate and folic acid in the periconceptional period: Recommendations from official health organizations in thirty-six countries worldwide and WHO. Public Health Nutr..

[37] Nilsen R.M., Vollset S.E., Gjessing H.K., Magnus P., Meltzer H.M., Haugen M., Ueland P.M. (2006). Patterns and predictors of folic acid supplement use among pregnant women: The Nowegian Mother and Child Cohort Study. Am. J. Clin. Nutr..

[38] Nilsen R.M., Leoncini E., Gastaldi P., Allegri V., Agostino R., Faravelli F., Ferrazzoli F., Finale E., Ghirri P., Scarano G. (2016). Prevalence and determinants of preconception folic acid use: An Italian multicenter survey. Ital. J. Pediatr..

[39] Peake J.N., Copp A.J., Shawe J. (2013). Knowledge and periconceptional use of folic acid for the prevention of neural tube defects in ethnic communities in the United Kingdom: Systematic review and meta-analysis. Birth Defects Res. A Clin. Mol. Teratol..

[40] Austin P.C. (2011). An introduction to propensity score methods for reducing the effects of confounding in observational studies. Multivar. Behav. Res..

[41] Martens E.P., Pestman W.R., Boer A.D., Belitser S., Klungel O.H. (2007). An important advantage of propensity score methods compared to logistic regression analysis. Pharmacoepidemiol. Drug Saf..

[42] Wissart J., Parshad O., Kulkarni S. (2005). Prevalence of pre- and postpartum depression in Jamaican women. BMC Pregnancy Childbirth.

[43] Choi H., Yamashita T., Wada Y., Kohigashi M., Mizuhara Y., Nagahara Y., Nishizawa S., Tominaga T., Fukui K. (2013). Predictors for exacerbation/improvement of postpartum depression—A focus on anxiety, the mothers’ experiences of being Cared for by their parents in childhood and borderline personality: A perspective study in Japan. J. Affect. Disord..

[44] Sugawara M., Toda M.A., Shima S., Mukai T., Sakakura K., Kitamura T. (1997). Premenstrual mood changes and maternal mental health in pregnancy and the postpartum period. J. Clin. Psychol..

